# Neutron imaging and modelling inclined vortex driven thin films

**DOI:** 10.1038/s41598-019-39307-x

**Published:** 2019-02-26

**Authors:** Timothy E. Solheim, Filomena Salvemini, Stuart B. Dalziel, Colin L. Raston

**Affiliations:** 1 Flinders Institute for NanoScale Science and Technology, College of Science and Engineering, Flinders University, Bedford Park, Adelaide, South Australia 5042 Australia; 2 Australian Centre for Neutron Scattering, ANSTO, Lucas Heights, New South Wales, 2234 Australia; 3 Department of Applied Mathematics and Theoretical Physics, University of Cambridge, Cambridge, CB3 0WA United Kingdom

## Abstract

The vortex fluidic device (VFD) is a thin film microfluidic platform which has a wide range of applications in synthesis and other areas of science, and it is important to understand the nature of the thin film of liquid in its inclined rapidly rotating tube. Neutron imaging has been used to determine the thickness of the film in a quartz tube with its shape modelled mathematically, showing good agreement between the model and experiments. The resultant equations are useful for studying VFD mediated processing in general, for which the optimal tilt angle of the tube is typically 45°. This includes its utility for the intelligent scale-up of organic syntheses, as demonstrated in the present study by the scaling up of an imine and amide synthesis to >1 g/min.

## Introduction

**Figure 1:**
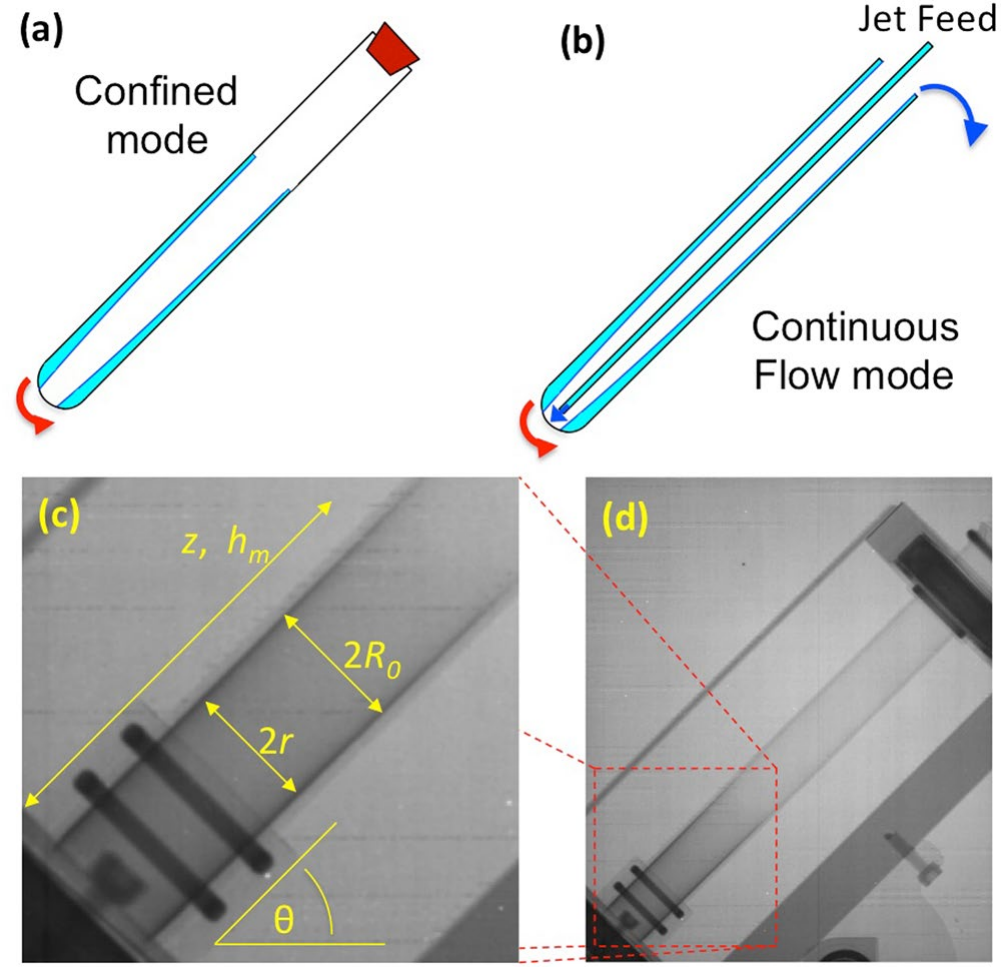
Illustrations of the set up for different modes of operation of the vortex fluidic device (VFD), (**a**) confined mode (CM) and (**b**) continuous flow (CF) mode, and neutron images of the VFD highlighting the mathematically important parameters, (**c** and **d**).

The vortex fluidic device (VFD) (Fig. 1) is a remarkably versatile thin film processing platform, which can operate under continuous flow conditions, where scalability of any process is considered at the inception of the science, as well as for small volumes in its so called confined mode^1^. It is able to refold proteins^1^, accelerate enzymatic and chemical reactions^2–4^, fabricate different nanocarbon materials^5,6^, incorporate drugs into bilayers, and a growing number of other applications. Despite these capabilities, optimisation of these processes is challenging without an understanding of the fluid behaviour within the VFD, particularly when attempting to scale the processes up to larger scales. This thin-film microfluidic platform features a hollow open-ended cylindrical tube (typically borosilicate glass or quartz with a 17.7 mm internal diameter) rapidly rotated at speeds, typically up to 9000 rpm. Liquid placed within the tube forms a thin film against the glass as a vortex to the bottom of the tube in an approximately a paraboloidal shape, and experiencing shear stresses on the order of 1 Pa^1^. The processing environment can be controlled by altering the tilt angle of the axis of the tube in relation to the horizontal (*θ*) position, the rotational speed (*ω*), and other operating parameters of the device (Fig. 1).

For the confined mode (CM) of operation of the VFD, a specific volume of fluid is placed within the tube, which is then capped and processed for a specified time^1,4,5,7^. Under continuous flow (CF), liquid is continuously delivered to the bottom of the tube through feed jets inserted at the top, or at strategic positions along it for multi-processing in a single VFD^7^. Once liquid in the film reaches the top of the tube, it exits the tube and is collected through a Teflon housing unit^2,6,8^. In general, for flow chemistry, the residence time is an important factor in controlling chemical and enzymatic reactions, and in controlling the formation of various carbon materials^9,10^. This often has a dramatic effect on the outcome of the processing, including in the VFD^2,8^. In most continuous flow systems, calculation of the residence time is straight-forward because the volume of the system is only affected by the number and size of the continuous flow processors. In contrast, the volume within the VFD is also affected by the rotational speed and tilt angle, causing the volume calculation to be more complicated. In order to calculate the residence time and better understand and design the processing, it is necessary to understand the shape of the film and how this is influenced by rotational speed, tilt angle, as well as the volume of liquid in the tube. The derivation of these equations allows quantitative answers to questions regarding the maximum volume the tube can hold in confined mode (without the liquid contacting the cap at the top of the tube which would perturb the film thickness and also the fluid dynamics), as well as the selection of operating conditions to give the desired residence time under continuous flow, allowing the intelligently-designed scale-up of chemical reactions.

## Results and Discussion

### Theoretical model

The model takes into account gravity and the centripetal force, and is consistent with previous derivations for Liquid Mirror Telescopes, which use the reflective paraboloid produced by mercury in a rotating container as an astronomical telescope^11,12^, as well as other work performed investigating the film shape^13^. The model is further detailed in the Supplementary Information, with the resultant equation for the film height

1 $$h=\frac{{\omega }^{2}}{2g\,\sin \,\theta }({r}^{2}-{R}_{0}^{2})+\sqrt{\frac{V}{{\rm{\pi }}}\frac{{\omega }^{2}}{g\,\sin \,\theta }},$$

which can be rearranged in the form for calculating the film thickness,

2 $${f}_{t}={R}_{0}-\sqrt{{R}_{0}^{2}+\frac{2g\,\sin \,\theta }{{\omega }^{2}}z-\frac{2}{\omega }\sqrt{\frac{gV\,\sin \,\theta }{{\rm{\pi }}}}}.$$

Here, *r* is the radial distance from the rotation axis of the tube and *z* is the distance along this axis above the base of the tube. The location of the film is influenced by the tube radius (*R*_0_), the rotational speed (*ω* rad/s), gravitational acceleration (*g*), the tilt angle (*θ*), and the volume of the fluid (*V*). The equations presented here are asymptotic approximations valid for high rotation rates (*ω*^2^*R*_*0*_ $\gg$ *g*) that, in the range of interest, agree with the full equations (presented in the SI) to better than 95%.

**Figure 2:**
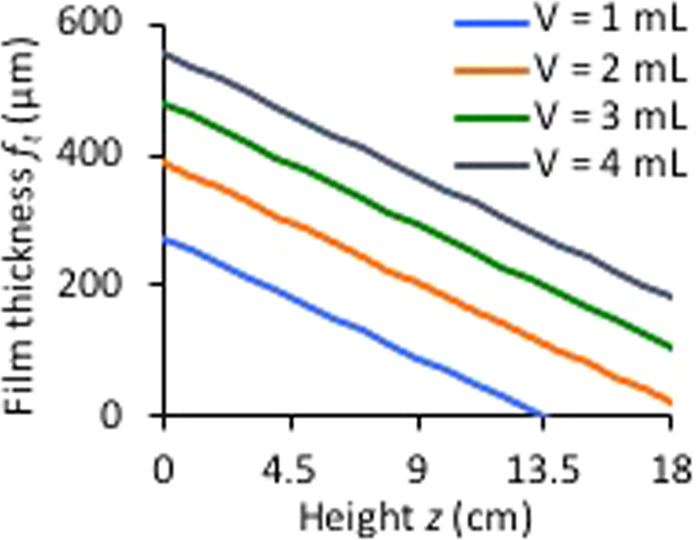
Mathematically determined film thickness in the VFD (confined mode) at different heights for various volumes of liquid (*V* = 1 mL, *θ* = 45°) (see Fig. 1 for definitions).

Under typical operating conditions of the VFD, with *R*_*0*_ = 8.75 mm (internal radius of a 20 mm OD tube), *θ* = 45° and *ω* = 6000 rpm (628 rad/s), the film is thin with *f*_*t*_ $\ll$ *R*_*0*_ and the thickness decreases almost linearly with height, Fig. 2, although the parabolic shape of the film is more pronounced when $\frac{g\,\sin \,\theta }{{\omega }^{2}{R}_{0}}$ is not too small at lower rotational speeds or when a variant of the device is used with *R*_*0*_ = 4.75 mm. In addition to determining the film thickness, it is important to know the maximum film height *h*_*m*_ because when this equals the height of the tube *H*, the volume of liquid in the VFD for CF is reached. When this volume is exceeded under CM, the liquid contacts the cap.

**Figure 3:**
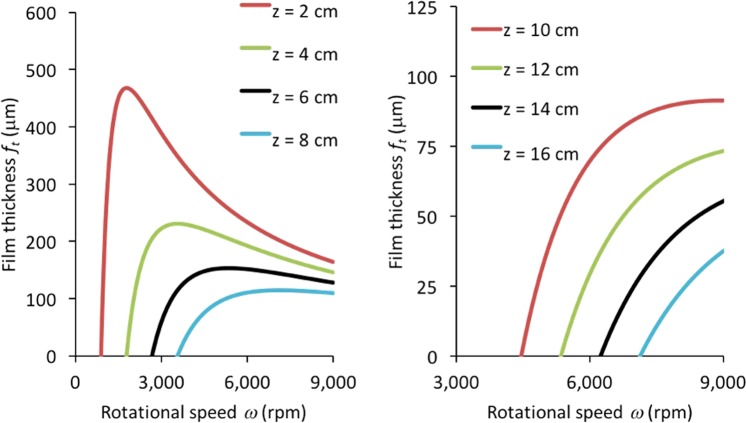
Calculated film thickness *f*_*t*_ as a function of rotational speed *ω* for various heights *z* of liquid in the VFD tube.

For applications of the VFD in chemical processing and in other areas of science, it is important to determine the conditions that result in the maximum film thickness for specific points along the tube. In considering the effect of rotational speed on film thickness for a particular height for a given volume of fluid, as depicted in Fig. 3, it is found that the film thickness initially exhibits a sharp increase, followed by a gradual decrease. This shape arises because the film thickness at a given height *z* starts at zero, when the rotation rate is still too low for the film to have reached this height. Then as the rotational speed increases, the film reaches this height producing a thin film. As the rotational speed is further increased, a thicker portion of the film is present at a given *z*, however the decrease in average film thickness partially counters and eventually overcomes this increase, leading to a decrease in the film thickness. Under particular operating parameters, it is important to maximise the film thickness at a particular height *z*, and thereby the rotational speed *ω*_*m*_ affording the greatest film thickness is given by

3 $${\omega }_{m}=2z\sqrt{\frac{g{\rm{\pi }}\,\sin \,\theta }{V}}.$$

This only applies for the CM operation of the device. For CF, the volume of fluid contained in the tube is a function of rotation rate. Hence an increase in rotational speed results in a reduction in the film thickness at all points due to the reduction in volume. It is interesting that the optimised rotational speed for laser-induced slicing of carbon nanotubes (7500 rpm) corresponds approximately to the rotational speed *ω*_*m*_ (7575 rpm) giving the maximum film thickness at the laser height (*z* = 8.5 cm).

An important result of the model is that there is a maximum volume *V*_*m*_ the device can contain under CM without it interacting with the cap at the top of the tube; this volume is also the volume within the device under CF mode of operation, and is given by

4 $${V}_{m}=\frac{g{H}^{2}{\rm{\pi }}\,\sin \,\theta }{{\omega }^{2}}.$$

The maximum volume (and hence the residence time *τ* under CF) is proportional to the inverse of the square of the rotational speed. This can be easily converted into an equation for the residence time *τ* based on the flow rate *Q*, using the equation *τ* = *VQ*^−1^.

### Application of the model

Previous work involving the optimisation of biodiesel synthesis using the VFD required experimentally measuring the residence time as a function of flow rate for *ω* = 6950 rpm (728 rad/s) at *θ* = 45°^14^. Re-analysing the data in the light of the present results indicates that a fit of *τ* = 158.57*Q*^−1^ results in a stronger correlation (as determined by the R^2^ correlation coefficient) than the originally reported fit of *τ* = 152.37*Q*^−0.934^ (R^2^ = 0.9894 compared to R^2^ = 0.9831 as originally reported). In comparing this experimental result to theoretical calculations, it is important to note that the experimentally determined residence time includes time spent within the Teflon collection unit at the top of the tube and the outlet pipette used to collect the liquid, which is not considered in the model. This gives the residence time as

5 $$\tau ={Q}^{-1}({V}_{E}+\frac{g{H}^{2}{\rm{\pi }}\,\sin \,\theta }{{\omega }^{2}}),$$

where *V*_*E*_ is the volume within the system that is external to the tube. Equating this to the experimentally determined formula of *τ* = 158.57*Q*^−1^ results in a *V*_*E*_ value of 1.16 mL, which is a reasonable estimate of the volume of the liquid residing in the Teflon unit and pipette. For a flow rate of 1 mL/min, the difference between this model and the previous correlation is less than 5%, but this relative difference is higher for lower flow rates (9% for 0.5 mL/min and 21% for 0.1 mL/min).

The developed model also provides insights into better understanding earlier processes performed within the VFD. Previously, the effect of rotational speed on the volume within the tube under continuous flow has been assumed to be minimal, in contrast to the model presented here. However, because increasing the rotational speed significantly decreases the volume within the tube, it is expected that a general decrease in yield may be observed with an increase in rotational speed for continuous flow processes due to the decreased residence time. This effect has been observed in previous esterification procedures, where it was instead attributed to evaporation of the solvent^2^. Additionally, it is revealed that for multi-step processing within a single tube, the residence time *τ*_*p*_ for a section from *z* = *z*_*1*_ to *z* = *z*_*2*_ is

6 $${\tau }_{p}=\frac{g{\rm{\pi }}\,\sin \,\theta }{Q{\omega }^{2}}({z}_{1}^{2}-{z}_{2}^{2}+2H({z}_{2}-{z}_{1})),$$

indicating that the calculation of overall residence time is more complicated than originally thought^8,15^.

### Neutron Imaging

The model was tested in developing an analytical technique to determine the film thickness *in situ*. Spectroscopic approaches were unsuccessful because the tapered nature of the thin film along the tube resulted in refraction, eliminating its potential for determining the film thickness. However, the unique imaging capabilities of neutrons enabled the measurement of both the film thickness and maximum height of the film extending up the tube.

Neutron imaging is a radiographic testing technique for viewing the interior of objects where the degree to which different elements attenuate (scatter or absorb) the probing neutron beam allows the visualisation of systems that are otherwise difficult or impossible to view by other methods. This is due to the particular physical properties of neutrons in comparison to other elementary particle probes. Neutrons are uncharged; they can deeply penetrate into matter and interact with the nucleus of an atom rather than with the diffuse electron cloud. Therefore, in neutron imaging techniques, the contrast is based on the ability of an element to attenuate neutrons, which is not linearly dependent on atomic number^16^. Consequently, air and water can be visually distinguished, despite having similar spectroscopic properties. Additionally, some metals (including those used in the bulk housing material of the VFD) are essentially transparent to neutrons, allowing easy subtraction of the VFD itself from the image.

**Figure 4:**
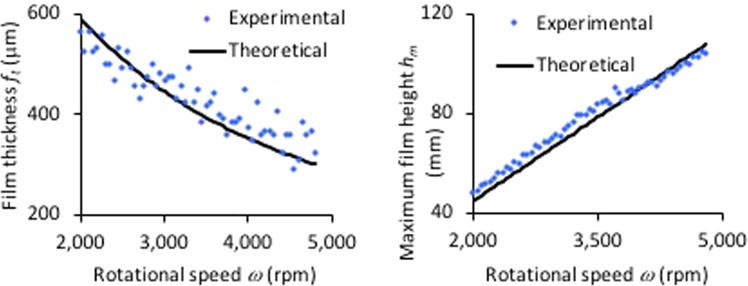
(**a**) Comparison of the mathematical model and neutron imaging determined film thickness in the VFD at various rotational speeds (*z* = (7.8 mm and 18.73 mm), *V* = 1 mL, *θ* = 45°). (**b**) Comparison of theoretical and experimental results for the maximum film height at various rotational speeds (*V* = 1 mL, *θ* = 45°) in the VFD.

The thin film produced in the VFD for 1 mL of water under confined mode at various rotational speeds was visualised under neutron imaging (3000 rpm shown in Fig. 1(c and d)). Close to the base of the tube is a pair of screws (in front and behind the quartz tube) that attach the VFD housing to the motor, and are not within the tube itself, despite appearing so in Fig. 1. Typically, a polycarbonate safety shield is placed around the rapidly rotating glass tube, but polycarbonate absorbs neutrons, and accordingly it was replaced by an aluminium shield which is essentially transparent to neutrons, appearing only as a thin dark grey line in Fig. 1(c). The medium grey sections of the image are the other much thicker aluminium components of the VFD. The black section in the top right is the top bearing (with the contrast from the lubricating grease), and similarly a black section for the lower bearing is noted in the bottom left corner. The VFD tube can be seen clearly, with the thin film appearing in black near the bottom of the tube, which is magnified in Fig. 1(c). The two black bands correspond to the two rubber O-rings that hold the VFD tube in place. After subtracting the image of the VFD devoid of any liquid present from the image containing some liquid, images such as those presented in the SI were obtained (Fig. S1), and the thickness and height of the film were measured for various rotational speeds (2000 rpm to 5000 rpm), with the 20.0 mm OD width of the tube used as a calibration. Speeds above this were impractical to measure accurately with the imaging as film thickness in the range of 400 μm corresponds to 6 pixels, and neither the film thickness nor the maximum height under these conditions were considered to be reliably determined. The measured film thickness, Fig. 4(a), shows reasonable correlation with the predicted values (R^2^ = 0.69), despite significant scatter due to the limited resolution for the film thickness measurements. In contrast, the maximum film height (shown in Fig. 4(b)) shows a good correlation (R^2^ = 0.95) with the values predicted from.

7 $${h}_{m}=\sqrt{\frac{V}{{\rm{\pi }}}\frac{{\omega }^{2}}{g\,\sin \,\theta }}.$$

Variations in the measured and predicted values are attributable to the errors associated with measuring film thickness of 10 pixels (667 μm) and less, as well as factors not considered in the model, such as surface tension and imperfections in the tube geometry. Future experiments are anticipated involving varying the volume and tilt angle, as well as changing the liquid and the nature of the surface of the tube, particularly the hydrophobic/hydrophilic balance, which is an important control parameter for optimising the outcome of VFD processing for a particular application^3^.

### Scaled-up imine and amide syntheses

Previous continuous flow operation of the VFD has involved a maximum flow rate of 2 mL/min for fast and often exothermic reactions, whereas typical flow rates are approximately 0.5 mL/min^2^. The ability to scale-up chemical reactions in the VFD is of interest in demonstrating the viability of the VFD for industrial scale processes. In order to allow increased flow rates without sacrificing residence time, it is necessary to increase the volume contained within the VFD. This was done using a special purpose constructed VFD housing a much longer 38 cm tube (compared to 18 cm for the standard VFD), with the same 20 mm O.D., and by minimising the rotational speed used. The minimum rotational speed capable of maintaining a vortex to the base of the glass tube is such that *f*_*t*_ = *R*_0_ at *z* = 0, giving $\omega ={R}_{0}^{-1}\sqrt{2gH\,\sin \,\theta }=2505\,{\rm{rpm}}$. In order to ensure a dry hemisphere at the base of the tube and prevent interaction between the feeds and the film, a slightly higher rotational speed of 3000 rpm was selected, which corresponds to a volume of 31.8 mL in the tube.

Previous conditions optimised for the synthesis of imines used a residence time of approximately 3 minutes^15^, which corresponds to a flow rate of 10 mL/min under the scaled-up design. The synthesis of three imines under this setup produced conversions of 82%, 87%, and 90% (Table 1, entries 1, 2 and 3), and purified yields of 82%, 45% and 83% after crystallisation. The conversions produced are nearly identical to those reported previously, demonstrating the successful scale-up to a twenty-fold production rate.

**Table 1:** The up-scaled synthesis of imines and amides in the VFD housing a standard 18 cm long glass tube, entries **1**–**3**, or a 38 cm long glass tube, entries **4**–**6** (both tubes 20 mm O.D.).

Entry	Compound	Conversion	Yield	Production rate (g/hr)
**1**		82%	82%	92
**2**		87%	45%	62
**3**		92%	83%	86
**4**		—	48%	38
**5**		—	26%	22
**6**		—	28%	22

Similarly, the previous synthesis of amides used a residence time of 40 seconds^17^, corresponding to a flow rate of 50 mL/min in the longer tube. However, the long-tube VFD operating at 3000 rpm was unable to maintain a thin film at this flow rate, resulting in the accumulation of fluid in the tube. Instead, a lower flow rate of 20 mL/min was selected, with the rotational speed increased to 4500 rpm to give the desired 40 seconds residence time and to produce a thinner film allowing better heat transfer. Under this system, yields of 48%, 26% and 28% were achieved for the three amides synthesised (Table 1, entries 4, 5, and 6). The lower yields in comparison to those previously reported may be because the thicker film (1480 μm at *z* = 0) will have reduced micromixing and shear stress, resulting in a less efficient reaction. Nevertheless, the yields remained higher in two of the three cases.

## Conclusions

In conclusion, we have developed a mathematical model describing the shape of the film in the VFD that is consistent with the experimentally determined film thickness as determined using neutron imaging. However, this novel approach has limitations in measuring film thickness at high rotational speeds of the VFD. Applying the model to previous work has elucidated the fact that under continuous flow, the increased residence time at low rotational speeds may result in higher yields than in the presence of pressure waves at high speeds. It was also determined that translating optimum conditions for the confined mode of operation of the device to continuous flow, a strategy which has served us well in some earlier research, may be an over simplification. The mathematical model was also used to design the scaled-up conditions for the synthesis of imines and amides, affording production rates ranging from 22 g/hour to 92 g/hour, which is a significant increase from previous procedures.

## Methods

### Neutron Imaging

Experiments were performed on the DINGO neutron beam line at the Australian Nuclear Science and Technology Organisation (ANSTO) OPAL research reactor at Lucas Heights, NSW, Australia. The instrument features a thermal neutron beam gathered through in-pile collimators positioned upstream of the beam line. In the case of real-time imaging of dynamic process, the inlet aperture of 20 mm in diameter (D) is configured to provide high flux and high spatial resolution (L/D of 500) at the length path (L) from the collimator to the sample position. The detector system was equipped with the CMOS camera (Andor NEO 5.5 sCMOS, 16 bit, 2560(w) × 2160(h) pixels sensor) coupled with 100 μm thick ZnS/^6^LiF scintillation screens. The field of view was adjusted to a nominal pixel size ≈ 67 μm by using a Carl Zeiss lens with a fixed focal length of 50 mm^18^. Exposure time for the camera was 1 s, with an additional 0.02 s of read out time required to transmit the data, resulting in a sampling frequency of 0.98 Hz. Initial investigations indicated that liquid within the VFD reached an equilibrium point after 5 seconds. The device was set to alternate between operation for 10 s and halt for 10 s, with each operation period increasing in rotational speed by 50 rpm. The device was operated at speeds from 2000 rpm to 4800 rpm. The raw images were processed using the program ImageJ, and 3 images were used to produce the normalised images; background signal *DF* (dark field, no beam), blank *OB* (open beam, neutron exposure and VFD, no liquid), and sample *I* (VFD with liquid, once equilibrium had been reached). Each image was preliminarily treated by applying a despeckle noise filter, and the normalised image *I*_*n*_ was produced by subtracting the background from both the blank and sample images, followed by dividing the sample image by the blank:

8 $${I}_{n}=\frac{I-DF}{OB-DF}.$$

Brightness and contrast of the corrected radiographies were then optimised to the grey level range of the region of interest with the liquid to produce images similar to that in Fig. S1a. Four points were used to measure film thickness, being the points at heights 0.78 cm and 1.873 cm, both closest to and furthest from the ground, as indicated by the blue dots in Fig. S1b.

### Scaled-up imine synthesis

55 mL solutions of amine and aldehyde were prepared in methanol at 1.94 M. The 38 cm long VFD was operated at 2970 rpm (due to technical issues of maintaining 3000 rpm for this modified VFD) and each solution was injected into the VFD at 5 mL/min for 10 minutes. The VFD was subsequently operated for an additional 3 minutes to allow all injected reagents to experience 3 minutes within the device. Output from the VFD was quenched in 500 mL of gently stirring water. Imines **1** and **2** formed an oil, and the water was split into 250 mL fractions, each washed with ethyl acetate (200 mL x3), recombined, dried (MgSO_4_), and the solvent removed under reduced pressure and *in vacuo* to give **1** as a yellow oil (15.9 g, with 3% residual aldehyde, giving yield of 15.5 g, 83%) and **2** as a solid. Compound **2** was subsequently vacuum filtered to give an impure yellow solid (21.1 g, 87%) which was recrystallised from ethyl acetate affording an off white solid (10.3 g, 45%). Compound **3** formed a solid, which was filtered, dissolved in ethyl acetate, dried (MgSO_4_), and the solvent removed under reduced pressure. Cooling gave an impure yellow solid (17.9 g, 92%) which was recrystallised form ethanol affording pale yellow crystals (14.6 g, 83%).

### Scaled-up amide synthesis

55 mL solutions of amine (0.43 M) in chloroform with 7% v/v triethylamine were prepared, along with 55 mL solutions of acylchloride (0.755 M) in chloroform. The VFD housing a 38 cm long glass tube was operated at 4500 rpm and each solution was injected into the VFD at 10 mL/min for 5 minutes. The VFD was subsequently operated for an additional 40 seconds to allow all injected reagents to experience 40 seconds within the device. Output from the VFD was quenched in 500 mL of gently stirring 2 M HCl. The organic layer was separated from the aqueous layer, dried (MgSO_4_), and solvent removed under reduced pressure, giving **4** as a solid and **5** and **6** as liquids. Amide **4** was vacuum filtered and recrystallised from ethanol affording a white solid (3.1 g, 48%). Amides **5** and **6** were dried *in vacuo* and recrystallised from ethanol affording **5** as a white solid (1.8 g, 26%), and **6** as a transparent solid (1.8 g, 28%).

## Characterisation

1. ^1^H NMR (600 MHz, CDCl_3_): δ 8.42 (s, 1 H), 7.82 (m, 2 H), 7.45–7.44 (m, 3 H), 7.37 (d ^3^*J*(H,H) = 4.4 Hz, 4 H), 7.31–7.28 (hex, ^3^*J*(H,H) = 4.2 Hz, 1 H), 4.86 (s, 2 H). ^13^C NMR (600 MHz, CDCl_3_): δ 162.1, 139.3, 136.2, 130.8, 128.7, 128.5, 128.3, 128.0, 127.0, 65.1. This is consistent with literature^14^.
2. ^1^H NMR (600 MHz, CDCl_3_): δ 8.99 (s, 1 H), 8.86 (d, ^3^*J*(H,H) = 9 Hz, 1 H), 7.89–7.88 (m, 3 H), 7.59–7.56 (m, 1 H), 7.51–7.49 (m, 2 H), 3.31 (tt, ^3^*J*(H,H) = 10.4 Hz, 4.1 Hz, 1 H), 1.87 (m, 4 H), 1.71 (m, 3 H), 1.44 (qt, ^3^*J*(H,H) = 12.4, 3.3, 2 H), 1.33 (qt, ^3^*J*(H,H) = 12.3, 3.5, 1 H). ^13^C NMR (600 MHz, CDCl_3_): δ 157.9, 133.8, 132.2, 131.4, 130.6, 128.6, 128.2, 126.9, 125.9, 125.3, 124.3, 70.9, 34.6, 25.8, 24.8. This is consistent with literature^14^.
3. ^1^H NMR (600 MHz, CDCl_3_): δ 8.47 (s, 1 H), 7.92–7.90 (m, 2 H), 7.49–7.47 (m, 3 H), 7.40 (t, ^3^*J*(H,H) = 7.8 Hz, 2 H), 7.25–7.21 (m, 3 H). ^13^C NMR (600 MHz, CDCl_3_): δ 160.4, 152.1, 136.2, 131.4, 129.2, 128.83, 128.79, 126.0, 120.9. This is consistent with literature^14^.
4. ^1^H NMR (600 MHz, CDCl_3_): δ 7.51 (d, ^3^*J*(H,H) = 7.9 Hz, 2 H), 7.31 (t, ^3^*J*(H,H) = 7.4 Hz, 2 H), 7.17 (bs, 1 H), 7.10 (t, ^3^*J*(H,H) = 7.4 Hz, 1 H), 2.34 (t, ^3^*J*(H,H) = 7.5 Hz, 2 H), 1.77 (hextet, ^3^*J*(H,H) = 7.4, 2 H), 1.01 (t, ^3^*J*(H,H) = 7.4 Hz, 3 H). ^13^C NMR (600 MHz, CDCl_3_): δ 171.2, 138.0, 129.0, 124.2, 119.8, 39.8, 19.1, 13.8. This is consistent with literature^19^.
5. ^1^H NMR (600 MHz, CDCl_3_): δ 7.34 (t, ^3^*J*(H,H) = 7.4 Hz, 2 H), 7.28–7.27 (m, 3 H), 5.67 (bs, 1 H), 4.45 (d, ^3^*J*(H,H) = 5.7 Hz, 2 H), 2.20 (t, ^3^*J*(H,H) = 7.5 Hz, 2 H), 1.70, (hextet, ^3^*J*(H,H) = 7.4 Hz, 2 H), 0.96 (t, ^3^*J*(H,H) = 7.4 Hz, 3 H). ^13^C NMR (600 MHz, CDCl_3_): δ 172.8, 138.4, 128.7, 127.9, 127.5, 43.6, 38.8, 19.2, 13.8. This is consistent with literature^17^.
6. ^1^H NMR (600 MHz, CDCl_3_): δ 5.26 (bs, 1 H), 3.77 (m, 1 H), 2.11 (t, ^3^*J*(H,H) = 7.5 Hz, 2 H), 1.92–1.90 (m, 2 H), 1.72–1.68 (m, 2 H), 1.65 (q, ^3^*J*(H,H) = 7.5 Hz, 2 H), 1.63–1.59 (m, 1 H + water), 1.36 (qt, ^3^*J*(H,H) = 12.5, 3.4 Hz, 2 H), 1.18–1.07 (m, 3 H), 0.94 (t, ^3^*J*(H,H) = 7.4 Hz, 3 H). ^13^C NMR (600 MHz, CDCl_3_): δ 172.0, 48.0, 39.1, 33.3, 25.6, 24.9, 19.3, 13.7. This is consistent with literature^20^.

## Supplementary information

Supplementary Information
