# Supplementary material for: Neutron imaging and modelling inclined vortex driven thin films
Source: Sci Rep. 2019 Feb 26;9:2817. doi: 10.1038/s41598-019-39307-x (PMC6391435; doi:10.1038/s41598-019-39307-x)
Supplement: Supplementary file 1 — Supplementary Information [file 41598_2019_39307_MOESM1_ESM.pdf]

Supplementary Information

Neutron imaging and modelling inclined vortex driven thin films

Timothy E. Solheim,[1] Filomena Salvemini,[2] Stuart B. Dalziel[3] and Colin L. Raston[1,*]

[1] Flinders Institute for NanoScale Science and Technology, College of Science and Engineering, Flinders University, Bedford Park, Adelaide, South Australia, 5042, Australia
[2] Australian Centre for Neutron Scattering, Lucas Heights, New South Wales, 2234, Australia
[3] Department of Applied Mathematics and Theoretical Physics, University of Cambridge, Cambridge, CB3 0WA, United Kingdom

Mathematical model – Force balance

There are a number of forces that operate within the Vortex Fluidic Device, including gravity, the centripetal force, electrostatic interactions between the liquid and the glass, viscous drag within the liquid, surface tension, and vibrations induced by imperfect rotation of the glass tube. Here, the net force $\vec{F}$ is considered to consist solely of the gravitational force $\overrightarrow{F_g}$ pulling the liquid down and the centrifugal force $\overrightarrow{F_c}$ pushing the liquid towards the walls of the tube, specifically

$$\vec{F} = \overrightarrow{F_g} + \overrightarrow{F_c}. \tag{S1}$$

The effect of surface tension is recognised as being not insignificant below the 'capillary length' $\left(\frac{\gamma}{\rho\omega^2 R_0}\right)^2 \sim O(100\mu m)$, where γ is the surface tension, ρ the fluid density, ω the angular frequency and R_0 the radius of the tube. However, their inclusion in the model would add unnecessary complexity, as the purpose of the model is to allow the calculation of useful parameters, rather than to gain a rigorous understanding of the fluid behaviour. Additionally, the chemical composition of the film is not uniform due to the conversion of reactants to products. Due to the high concentrations often used within the device, this conversion results in a variation in temperature, viscosity, and surface tension throughout the film. If viscosity and surface tension were considered in the model, this non-uniformity would need to be considered, and would result in a highly complicated model.

In considering the tilt angle, two approaches to selecting the coordinate system can be taken: using a coordinate system that remains fixed relative to the direction of gravity, or using a system that tilts with the tube. Here the latter approach has been taken. We align the *z*-axis with the rotation axis of the tube. The tilt angle θ is then defined such that $\theta = 0$ corresponds to gravity, *g*, acting in the –*x* direction $(-g, 0, 0)$ (corresponding to the VFD tube positioned horizontally with the base on the right), and $\theta = \frac{\pi}{2}$ corresponds to gravity acting downwards $(0, 0, -g)$ (VFD positioned vertically). The force on the fluid per unit volume due to gravity is therefore

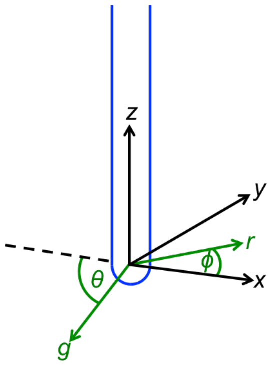

$$\overrightarrow{F_g} = -\rho g(\cos\theta, 0, \sin\theta). \tag{S2}$$

The centrifugal force per unit volume is

$$\overrightarrow{F_c} = \rho r\omega^2(\cos\phi, \sin\phi, 0), \tag{S3}$$

where *r* is the radial distance from the rotation (*z*) axis. Here, ϕ is the angular position around the tube in the *x*-*y* plane, such that $\phi = 0$ corresponds to the *x* axis and $\phi = \frac{\pi}{2}$ corresponds to the *y* axis. The net force per unit volume on the fluid is therefore

$$\vec{F} = \rho(r\omega^2\cos\phi - g\cos\theta, r\omega^2\sin\phi, -g\sin\theta). \tag{S4}$$

Mathematical Model – Determining the shape

The system is considered only when in equilibrium, and consequently at the free surface the net force per unit volume must be exerted in a direction orthogonal to the surface of the liquid. We define the surface of the film by $z = h(x, y)$, or equivalently $z - h(x, y) = 0$. The vector normal to the surface, $\vec{n}$, is therefore

$$\vec{n} = \nabla\big(z - h(x, y)\big) = \left(-\frac{\partial h}{\partial x}, -\frac{\partial h}{\partial y}, 1\right). \qquad \text{(S5)}$$

The direction of the net force must be parallel to $\vec{n}$ and so $\vec{F}\times\vec{n} = \vec{0}$. Substituting (S4) and (S5) into the vector cross product reveals

$$\frac{\partial h}{\partial y} = \frac{r\omega^2 \sin\phi}{g \sin\theta}, \qquad \frac{\partial h}{\partial x} = -\frac{\cos\theta}{\sin\theta} + \frac{r\omega^2 \cos\phi}{g \sin\theta}. \qquad \text{(S6)}$$

It is more useful, however, to use the chain rule to perform a change of variable from $h(x, y)$ to $h(r, \theta)$ and note that

$$\frac{\partial h}{\partial r} = \frac{\partial h}{\partial x}\frac{\partial x}{\partial r} + \frac{\partial h}{\partial y}\frac{\partial y}{\partial r}. \qquad \text{(S7)}$$

Since $x = r\cos\theta$ and $y = r\sin\theta$, we recover

$$\frac{\partial h}{\partial r} = \frac{r\omega^2}{g \sin\theta} - \cos\phi \cot\theta. \qquad \text{(S8)}$$

Integrating (S8) then gives the height

$$h = \frac{r^2\omega^2}{2g \sin\theta} - r\cos\phi\cot\theta + k, \qquad \text{(S9)}$$

where k is a constant of integration that is yet to be determined.

Mathematical model – Determination of k from the volume

The constant of integration k can be determined from the volume V of fluid in the system. Specifically,

$$V = \int_0^{2\pi}\int_{r_i}^{R_0} hr\, dr\, d\phi, \qquad \text{(S10)}$$

where r_i is the radius at which the film intersects the base of the tube, or zero if the fluid covers the entire base. For a flat-bottomed tube, we thus solve $h = 0$ for r_i and obtain

$$r_i = A\cos\phi + \sqrt{A^2\cos^2\phi + B}, \qquad \text{(S11)}$$

where

$$A = \frac{g\cos\theta}{\omega^2}, B = -\frac{2g\sin\theta}{\omega^2}k. \qquad \text{(S12)}$$

Computing the inner integral of (S10) is straight forward, giving

$$V = \int_0^{2\pi}\left(\frac{(R_0^4 - r_i^4)\omega^2}{8g\sin\theta} - \left(\frac{R_0^3 - r_i^3}{3}\right)\cos\phi\cot\theta + \frac{1}{2}(R_0^2 - r_i^2)k\right)d\phi. \qquad \text{(S13)}$$

Substituting for r_i using (S12) then integrating reduces this to

$$V = \frac{\pi}{4}\left(\frac{g^3\cos^4\theta}{\omega^6\sin\theta}\right) - \pi\left(\frac{g^2\cos^2\theta}{\omega^4}k\right) + \pi\left(\frac{gk^2\sin\theta}{\omega^2} + \frac{R_0^4\omega^2}{4g\sin\theta} + R_0^2 k\right). \qquad \text{(S14)}$$

We can now select k in one of two ways. In particular, we can specify the volume V and solve (S14) for k, or we can specify k such that the fluid just reaches the top of the tube at

$\phi = \pi$, noting that $\phi = \pi$ is the angular position giving the greatest h. The first of these is appropriate for confined mode, while the second describes the VFD under continuous flow. The first of these yields

$$h = \frac{\omega^2}{2g\sin\theta}(r^2 - R_0^2) - r\cos\phi\cot\theta - \frac{g\cos^2\theta}{2\omega^2\sin\theta} + \sqrt{\frac{1}{2}R_0^2\cot^2\theta + \frac{\omega^2}{g\pi\sin\theta}V}, \tag{S15}$$

which is valid only provided $h_m = h(r = R_0, \phi = \pi) \le H$, where H is the length of the tube and

$$h_m = R_0\cot\theta - \frac{g\cos^2\theta}{2\omega^2\sin\theta} + \sqrt{\frac{1}{2}R_0^2\cot^2\theta + \frac{\omega^2}{g\pi\sin\theta}V}. \tag{S16}$$

Having derived (S15), various useful quantities can be derived. Rearranging (S15) gives the film thickness f_t as a function of the height of interest z as

$$f_t = R_0\left(1 - \frac{g}{\omega^2 R_0}\cos\phi\cos\theta - \sqrt{1 + \left(\frac{g}{\omega^2 R_0}\right)^2\cos^2\theta\,(1+\cos^2\phi) + 2\left(\frac{g}{\omega^2 R_0}\right)\frac{z}{R_0}\sin\theta - \sqrt{4\frac{g}{\omega^2 R_0}\frac{V}{R_0^3}\frac{\sin\theta}{\pi} + 2\left(\frac{g}{\omega^2 R_0}\right)^2\cos^2\theta}}\right) \tag{S17}$$

The maximum volume V_m a tube of height H can hold is determined by setting $h_m = H$ and solving for the volume,

$$V_m = \pi H R_0^2\left(\frac{g}{\omega^2 R_0}\left(\frac{H}{R_0}\sin\theta - 2\cos\theta + \frac{1}{2}\frac{R_0}{H}\frac{\cos^2\theta}{\sin\theta}\right) + 2\left(\frac{g}{\omega^2 R_0}\right)^2\cos^2\theta\left(1 - \frac{R_0}{H}\cot\theta\right) - \frac{1}{4}\left(\frac{g}{\omega^2 R_0}\right)^3\frac{R_0}{H}\frac{\cos^4\theta}{\sin\theta}\right). \tag{S18}$$

Derivations of the equations in the paper

It is important to know the full equations in order to gain a complete understanding of the theory. However, when applying the equations in practice, their simplification to a shorter form is preferable, even at the expense of a small loss of accuracy. In all cases, the approximation used is that the component of gravity in the radial direction is negligible, which is justified provided it is small compared to the magnitude of the centripetal force, specifically $\varepsilon \ll 1$, where $\varepsilon = \frac{g}{\omega^2 R_0}$, with typical operating conditions giving $\varepsilon \sim O(10^{-2})$. This approximation results in a symmetric film. Under this approximation, the equations presented in the paper are produced from (S16), (S15) and (S17), giving

$$h = \frac{\omega^2}{2g\sin\theta}(r^2 - R_0^2) + \sqrt{\frac{V}{\pi}\frac{\omega^2}{g\sin\theta}}, \tag{1}$$

$$f_t = R_0 - \sqrt{R_0^2 + \frac{2g\sin\theta}{\omega^2}z - \frac{2}{\omega}\sqrt{\frac{gV\sin\theta}{\pi}}}\,, \tag{2}$$

$$h_m = \sqrt{\frac{V}{\pi}\frac{\omega^2}{g\sin\theta}}. \tag{3}$$

The calculation of some useful quantities is more easily performed using the $\varepsilon \ll 1$ simplification, including the rotational speed ω_m giving the greatest film thickness for a particular z location. This requires $\frac{df_t}{d\omega} = 0$, giving

$$\omega_m = 2z\sqrt{\frac{g\pi\sin\theta}{V}} \tag{4}$$

The approximation also produces a simpler equation for the maximum volume within the tube from (S18),

$$V_m = \frac{gH^2\pi\sin\theta}{\omega^2}. \tag{5}$$

The residence time τ is calculated by dividing the volume by the flow rate Q. Here, the actual volume is $V_E + V_m$, where V_E is the volume in the system external to the tube, that is contained within the Teflon housing unit and the dropping pipette. Using (5) to calculate the volume, this gives a residence time of

$$\tau = Q^{-1}(V_E + V_m) = Q^{-1}\left(V_E + \frac{gH^2\pi\sin\theta}{\omega^2}\right). \tag{6}$$

In addition to calculating the total residence time, it is often useful to know the residence time τ_p for a particular section of the tube under continuous flow. This requires calculation of the volume of liquid in the tube in any given section. This allows a relatively straightforward determination of the volume in a particular section from z_1 to z_2 as

$$\tau_p = Q^{-1}\int_{z_1}^{z_2}\pi(2R_0f_t - f_t^2)\,dz = \pi Q^{-1}\left(-g(z_2^2 - z_1^2)\frac{\sin\theta}{\omega^2} + (z_2 - z_1)\sqrt{\frac{4gV_m\sin\theta}{\pi\omega^2}}\right).$$

When considered under continuous flow, this produces

$$\tau_p = \frac{g\pi\sin\theta}{Q\omega^2}\left(z_1^2 - z_2^2 + 2H(z_2 - z_1)\right). \tag{7}$$

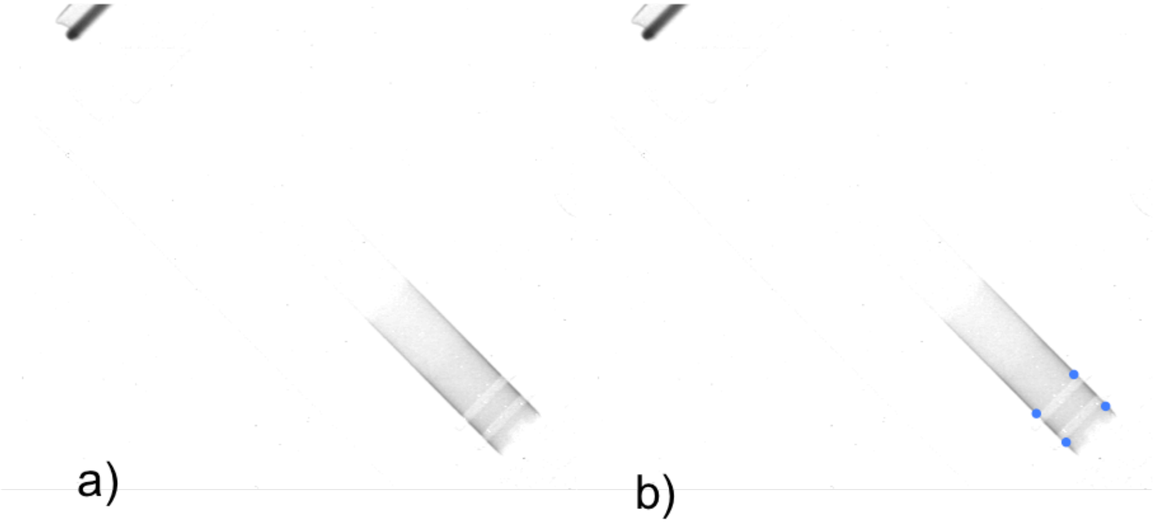

Fig. S1. a) Typical image produced from Neutron Imaging experiments. b) Same image, but with blue dots denoting film thickness measurement locations.

Notes on typical spectroscopy attempts

Typical spectroscopy proved unviable for determining the film thickness because the slope of the film results in scattering of the light source, an effect that does not occur in the case of an empty tube. Consequently, the “absorbance” read by the detector is largely from scattering.

Characterisation

1 – ^{1}H NMR

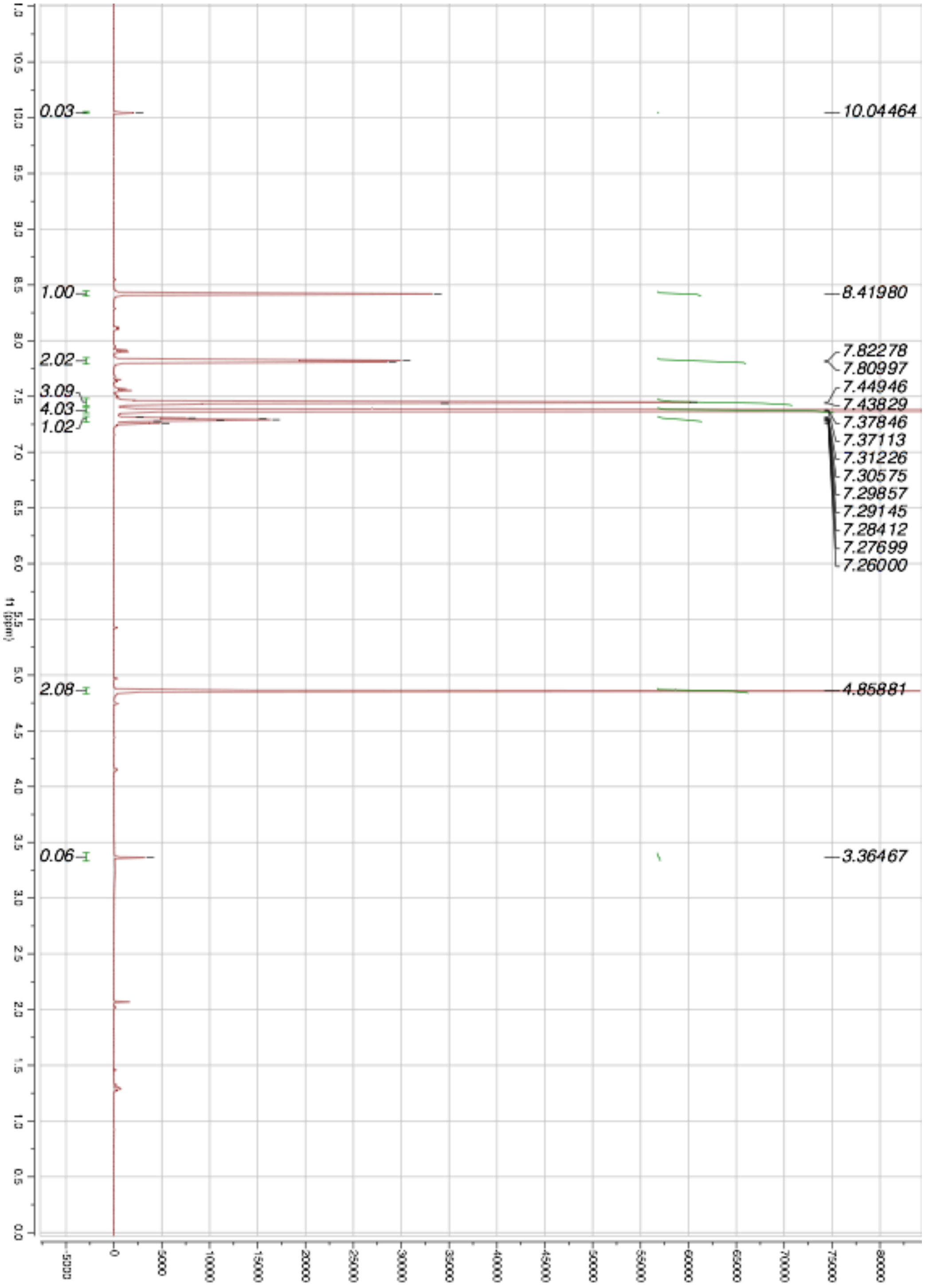

The singlets at 10.04 and 3.36 ppm indicate the presence of trace amounts of benzaldehyde and benzylamine respectively, and are taken into account in the yield calculation.

1 – ^{13}C NMR

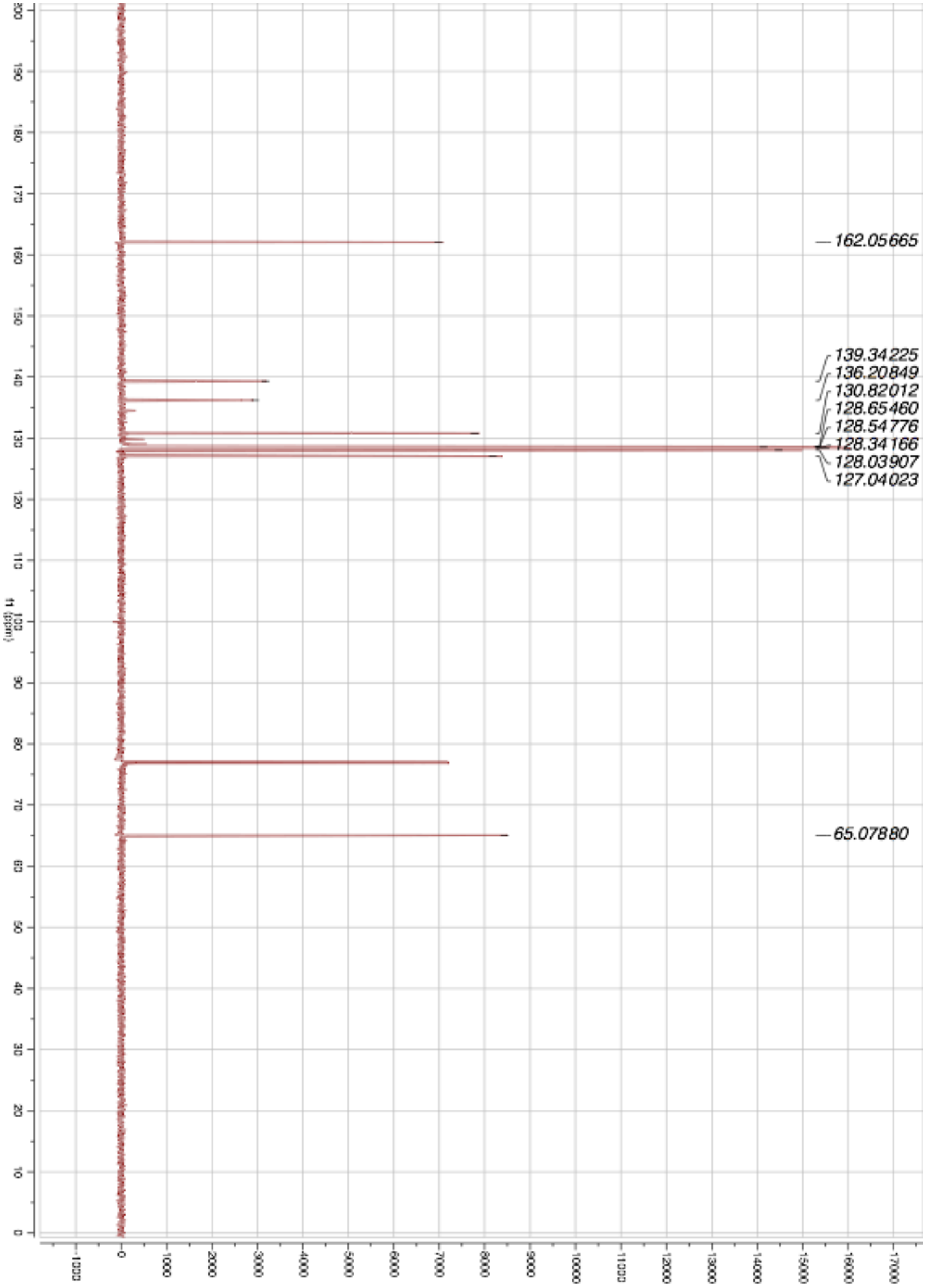
162.05665
139.34225
136.20849
130.82012
128.65460
128.54776
128.34166
128.03907
127.04023
65.07880
f1 (ppm)

2 – ^{1}H NMR

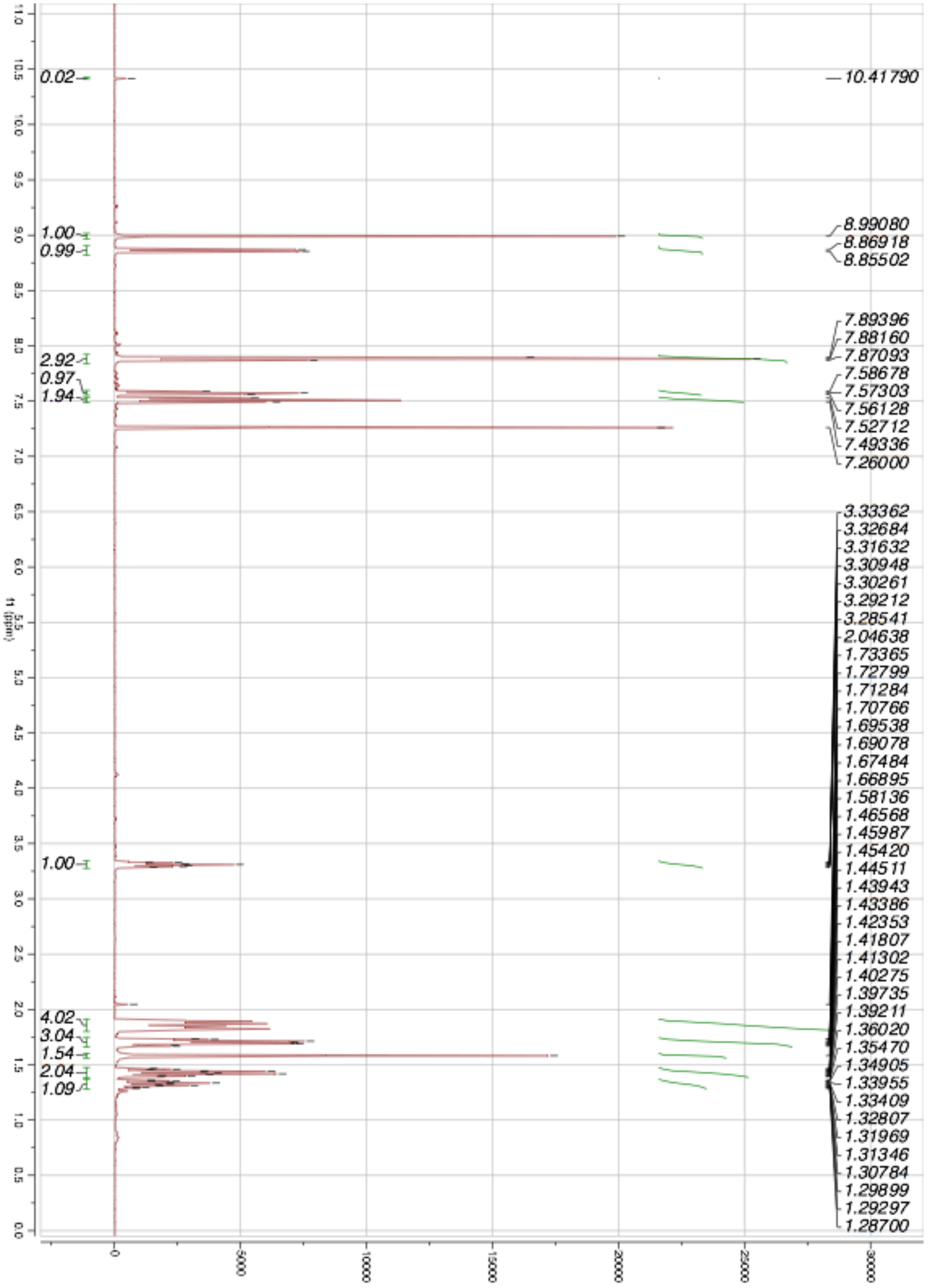

The singlet at 10.42 ppm indicates the presence of trace amounts of residual 1-naphthaldehyde, and the broad peak at 1.58 ppm is from by HDO in the NMR solvent.

2 – ^{13}C NMR

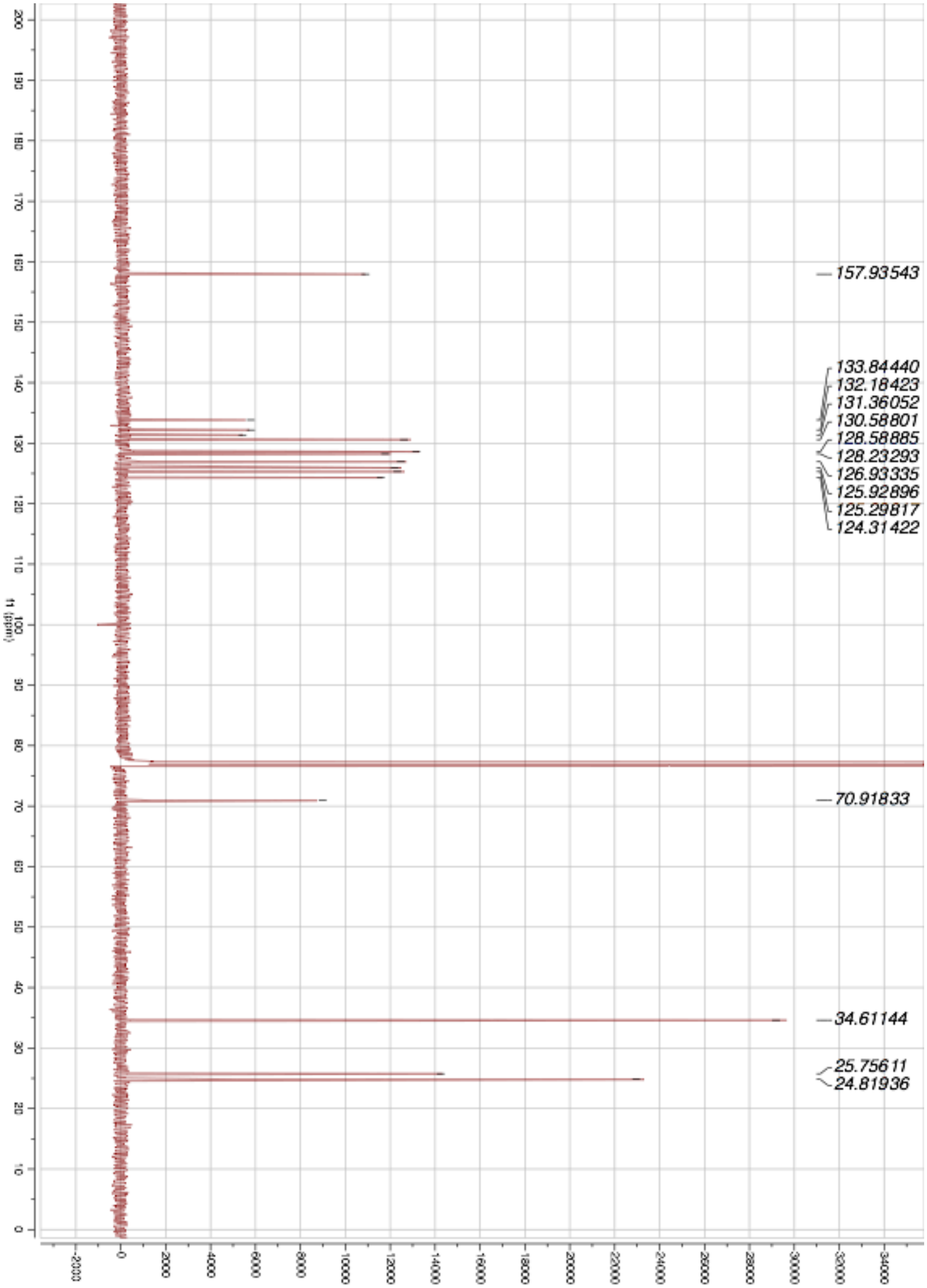

3 – ^{1}H NMR

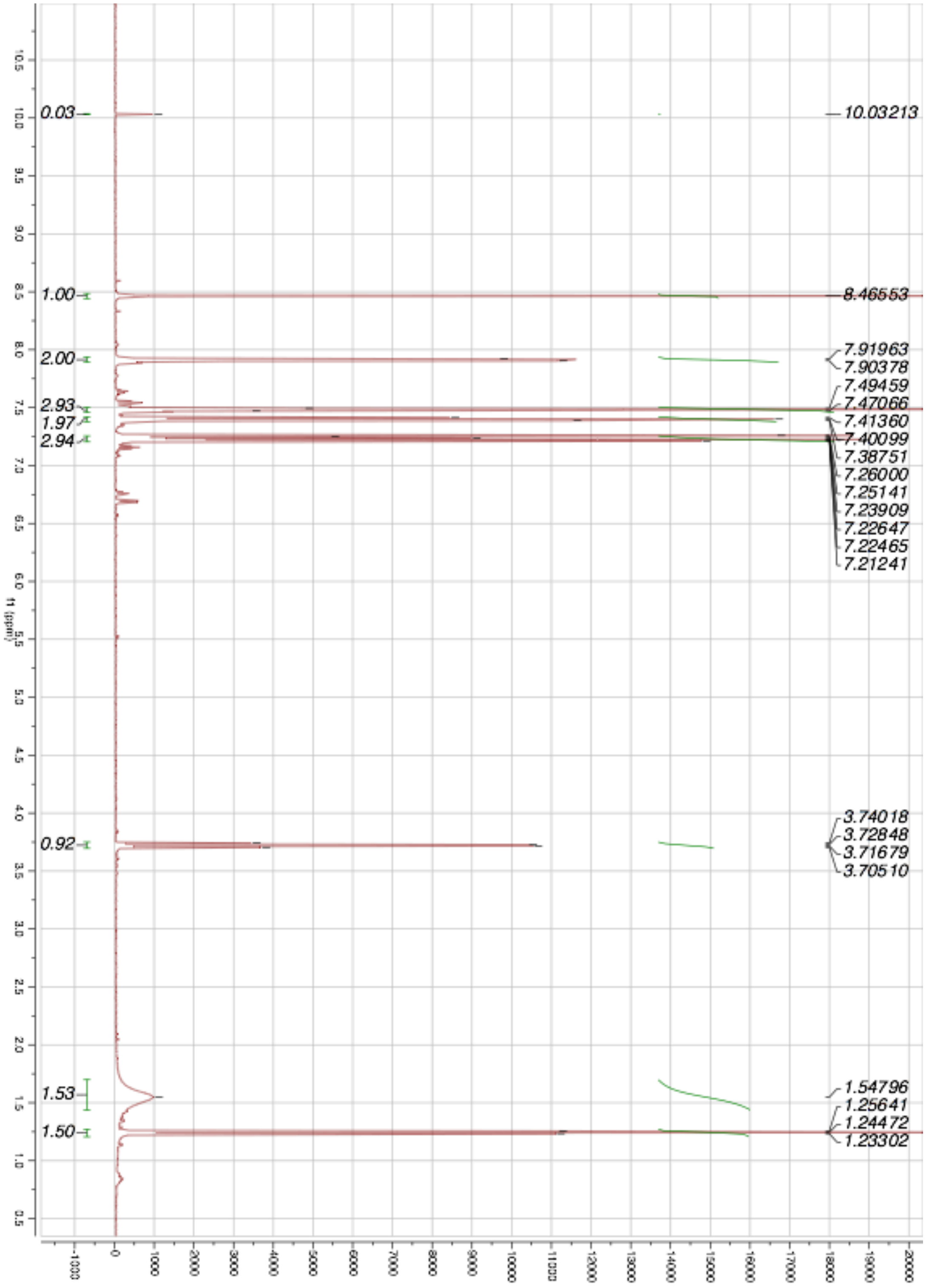

The singlet at 10.03 ppm indicates trace amounts of residual benzaldehyde. The peaks at 3.72, 1.55 and 1.24 ppm indicate the presence of residual ethanol from recrystallization (at 11% w/w), which is taken into account in the yield calculation.

3 – ^{13}C NMR

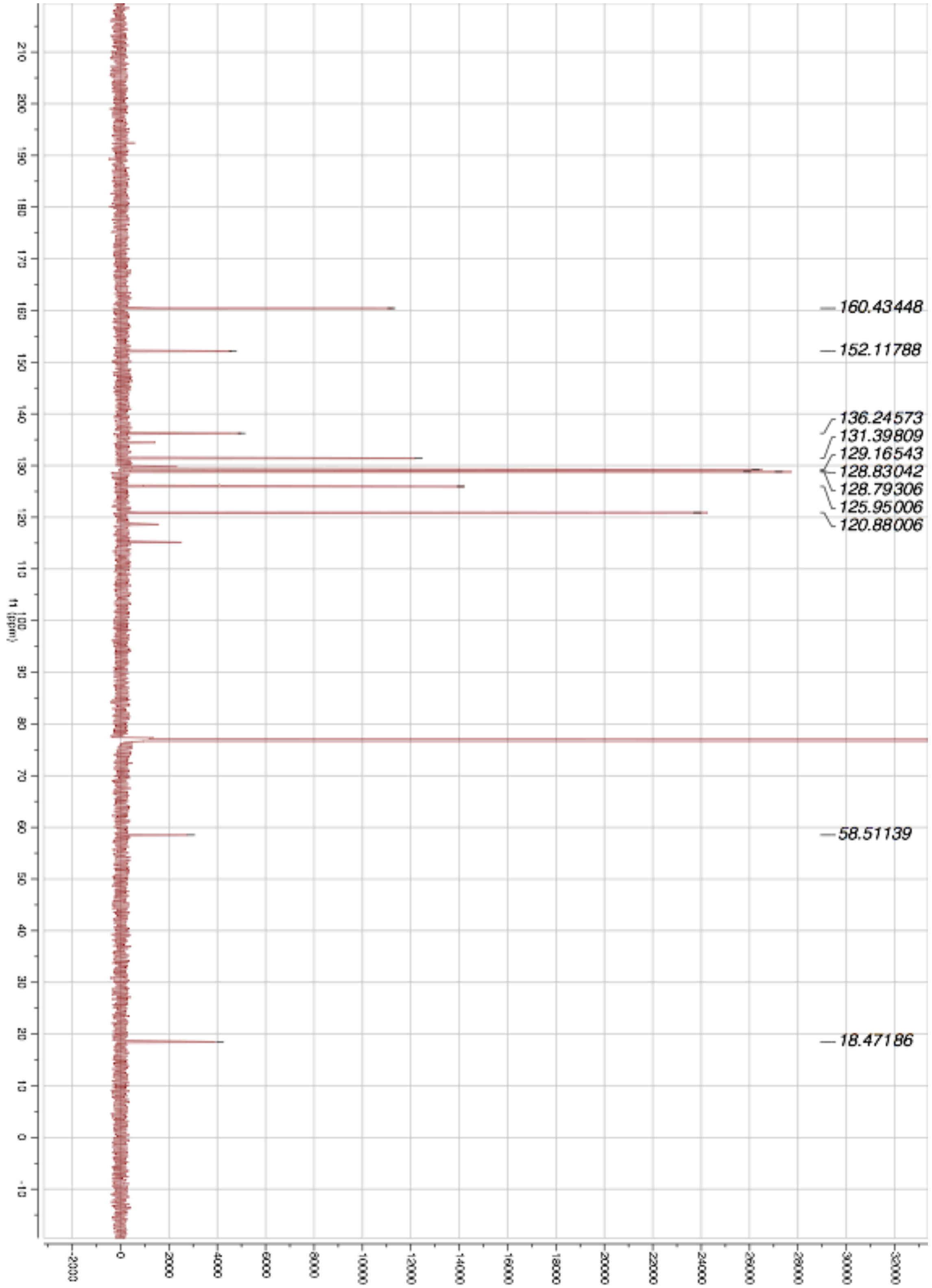

The peaks at 58.5 and 18.5 ppm are caused by the residual ethanol as noted above.

4 – ^{1}H NMR

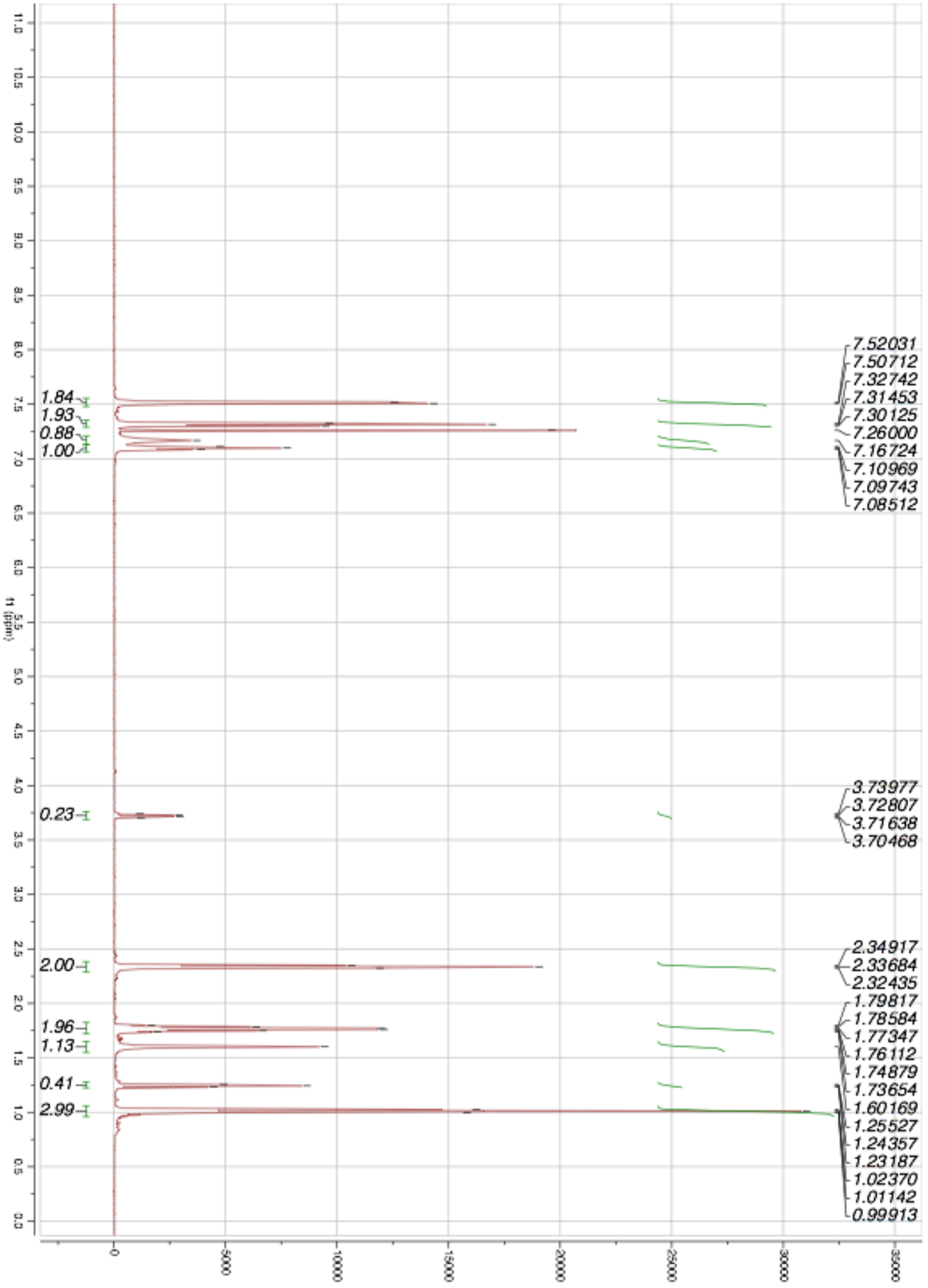

The peaks at 3.72 and 1.24 ppm indicate the presence of residual ethanol from the recrystallization (at 3% w/w), which is taken into account in the yield calculation. The peak at 1.60 ppm is from by HDO in the NMR solvent.

4 – ^{13}C NMR

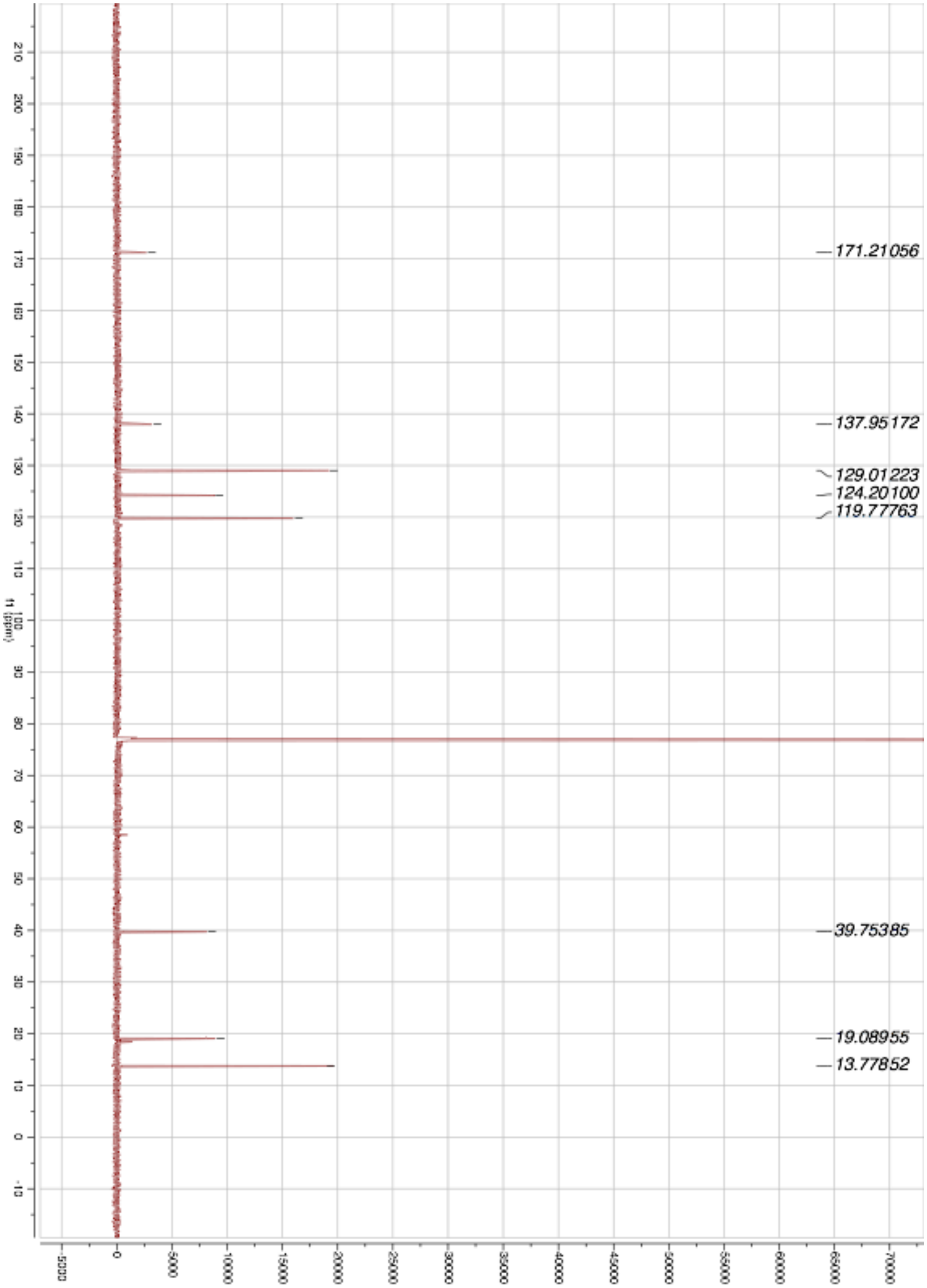
171.21056
137.95172
129.01223
124.20100
119.77763
39.75385
19.08955
13.77852
f1 (ppm)

5 – ^{1}H NMR

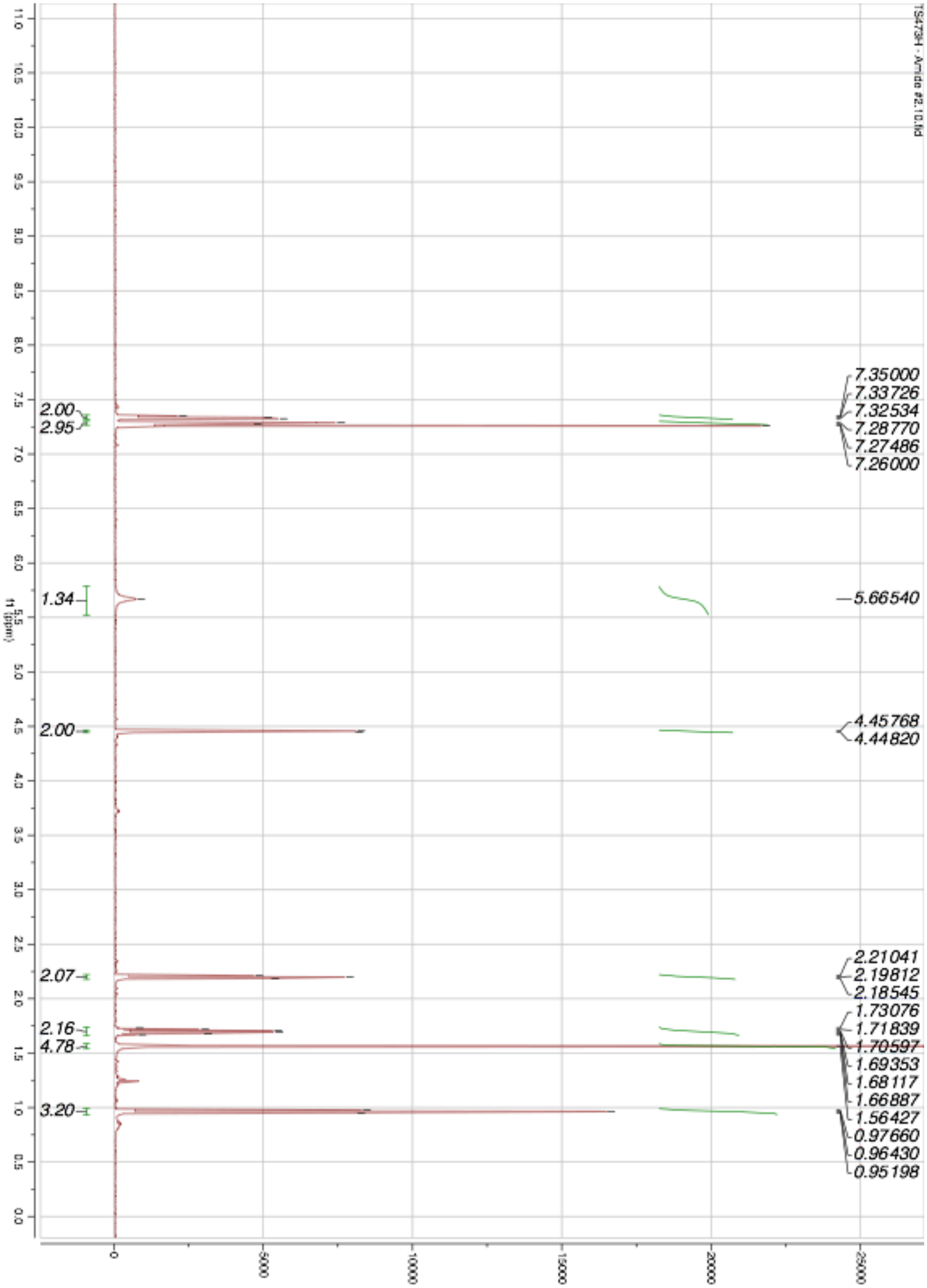

The peak at 1.56 ppm is from by HDO in the NMR solvent.

5 – ^{13}C NMR

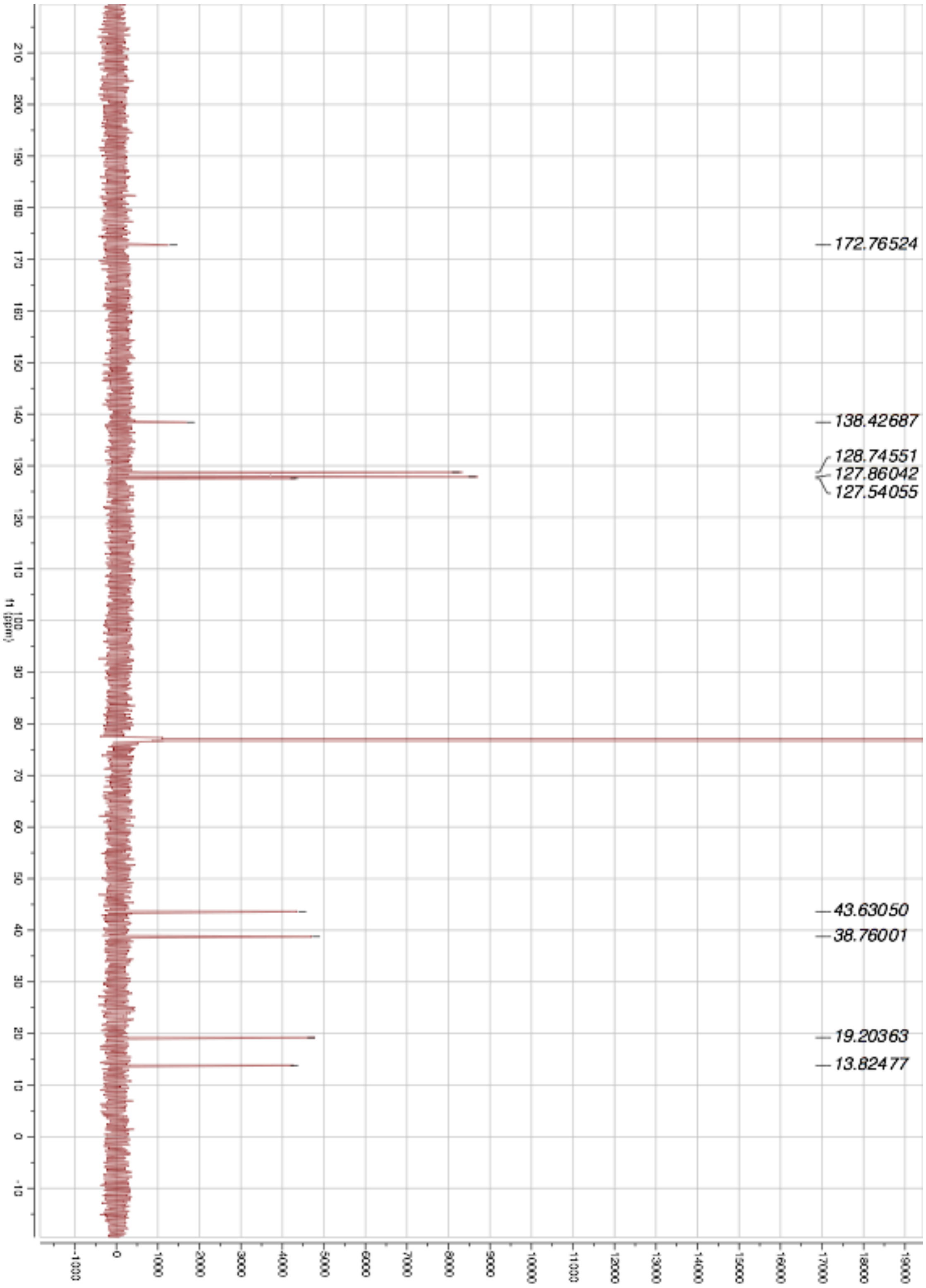

6 – ^{1}H NMR

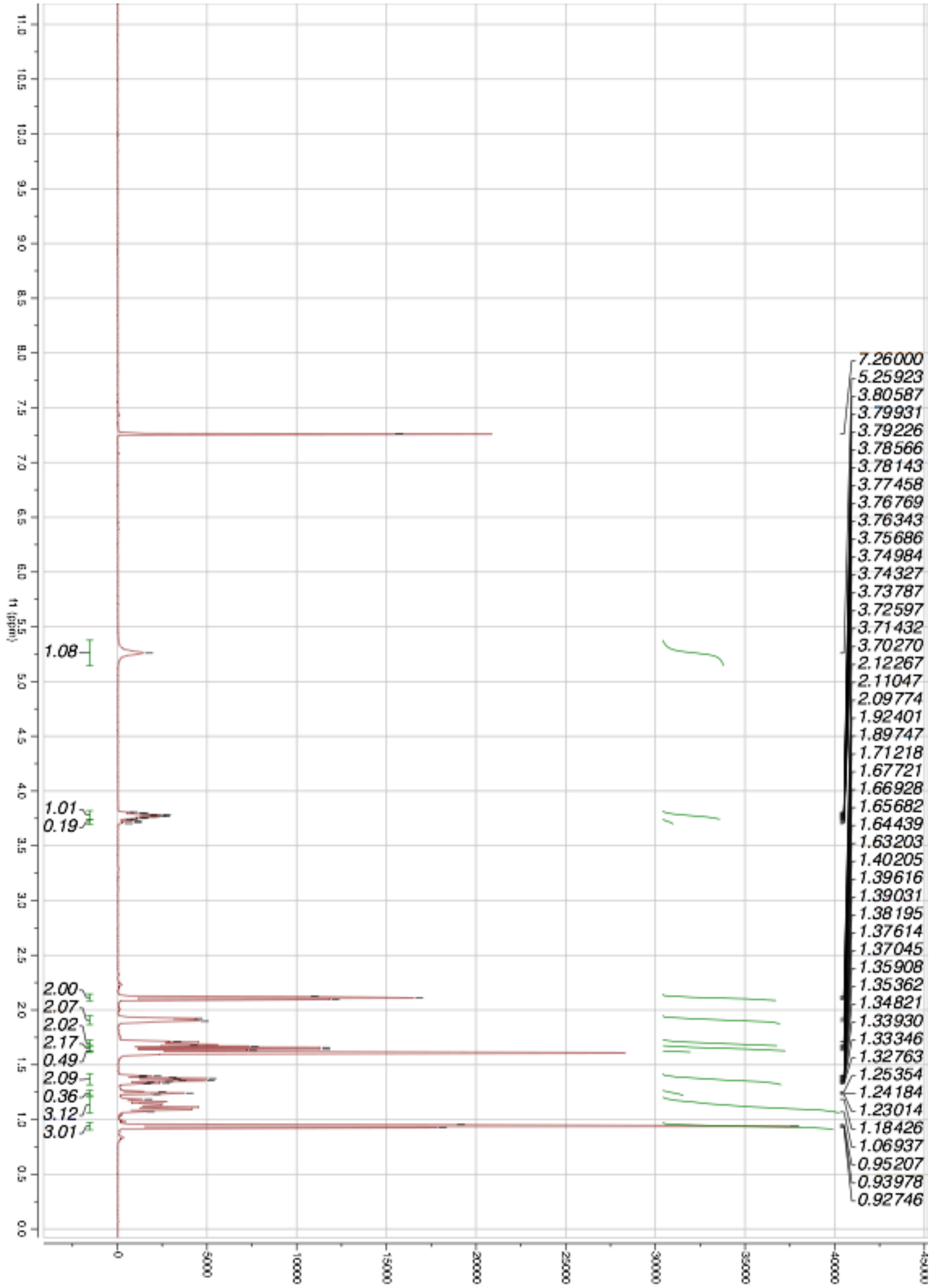

The peaks at 3.72 and 1.24 ppm indicate the presence of residual ethanol from the recrystallization (at 3% w/w), which is taken into account in the yield calculation. The peak at 1.60 ppm is from by HDO in the NMR solvent and overlaps a multiplet.

6 – ^{13}C NMR

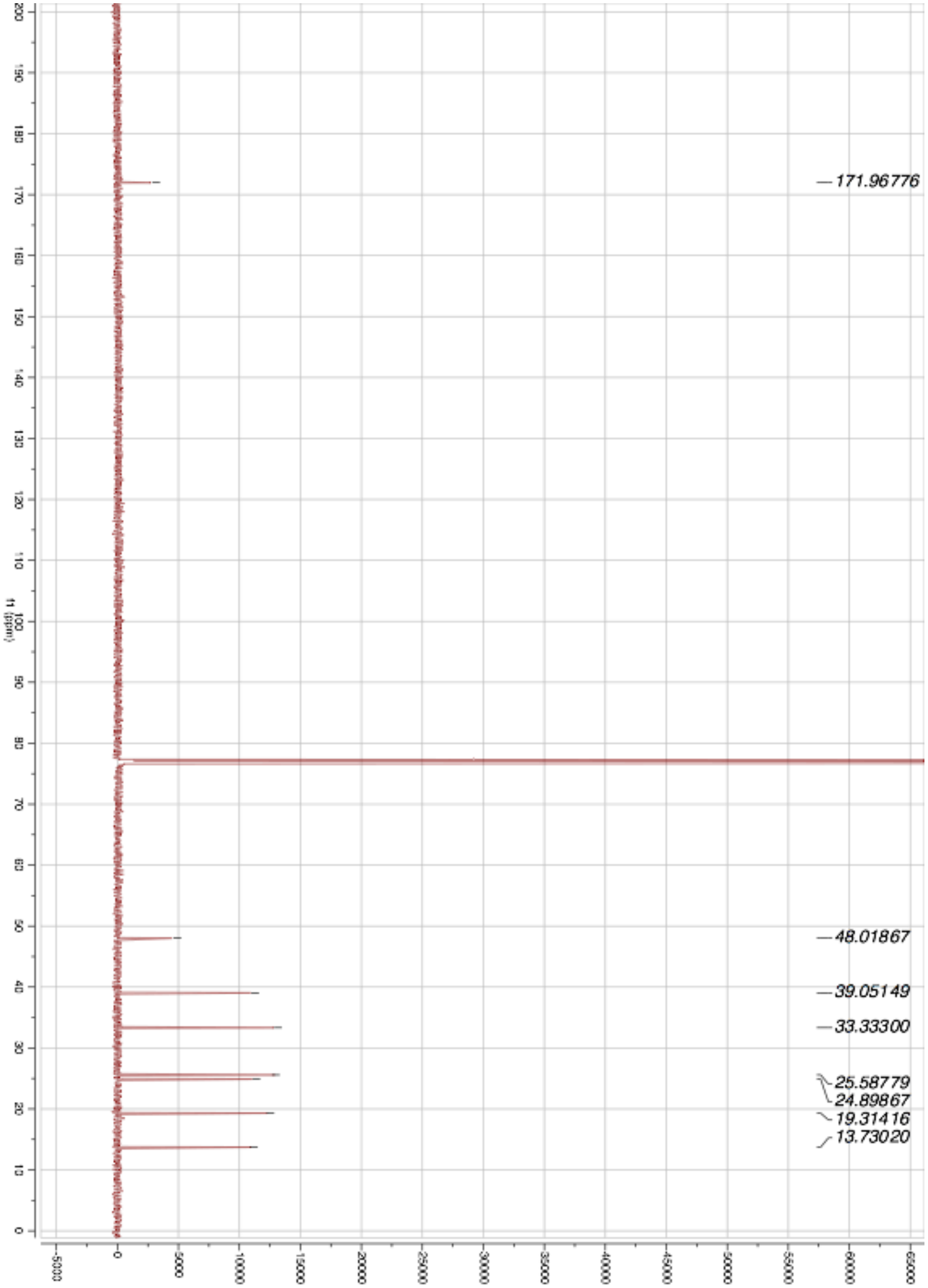
171.96776
48.01867
39.05149
33.33300
25.58779
24.89867
19.31416
13.73020
f1 (ppm)
